# T2DM patients with depression have higher levels of hyperglycemia and cognitive decline than T2DM patients

**DOI:** 10.1371/journal.pone.0273327

**Published:** 2022-08-19

**Authors:** Savitree Thummasorn, Sopida Apichai, Supat Chupradit, Pornpen Sirisattayawong, Pachpilai Chaiwong, Sirawit Sriwichaiin, Wasana Pratchayasakul, Nipon Chattipakorn, Siriporn C. Chattipakorn

**Affiliations:** 1 Department of Occupational Therapy, Faculty of Associated Medical Sciences, Chiang Mai University, Chiang Mai, Thailand; 2 Neurophysiology Unit, Cardiac Electrophysiology Research and Training Center, Faculty of Medicine, Chiang Mai University, Chiang Mai, Thailand; 3 Center of Excellence in Cardiac Electrophysiology Research, Chiang Mai University, Chiang Mai, Thailand; 4 Department of Oral Biology and Diagnostic Sciences, Faculty of Dentistry, Chiang Mai University, Chiang Mai, Thailand; Essen University Medical School, GERMANY

## Abstract

The cognitive impairment, depression, a decrease in the ability to perform activities of daily living (ADLs), and salivary gland dysfunction, as indicated by the reduction of alpha-amylase activity, have been reported in patients with type 2 diabetes (T2DM). However, the effects of depression on cognitive function, salivary alpha-amylase activity, and ADLs in T2DM patients have never been investigated. In this study, 115 participants were divided into three groups, including 30 healthy people, 50 T2DM patients without depression, and 35 T2DM patients with depression. Then, the cognitive function, the level of depression, salivary-alpha amylase activity, ADLs, and metabolic parameters were determined. Results showed that T2DM patients had hyperglycemia and cognitive impairment. A decrease in the salivary alpha-amylase activity was observed in T2DM patients. Interestingly, T2DM patients with depression had higher level of hyperglycemia and cognitive impairment than T2DM patients. Additionally, cognitive function was associated with the salivary-alpha amylase activity in T2DM without depression, while the severity of depression was associated with the salivary-alpha amylase activity in T2DM patients with depression. Therefore, we concluded that T2DM caused the impairment of metabolism, decreased salivary alpha-amylase activity, and cognitive impairment. Furthermore, T2DM patients with depression had higher level of hyperglycemia and cognitive decline than T2DM patients.

## Introduction

It has been established that type 2 diabetes mellitus (T2DM) causes several complications, such as cardiovascular disease, systemic inflammation, and neurodegenerative disease [1–3]. Previous studies have demonstrated that T2DM is a risk factor for mild cognitive impairment (MCI) [4], increased learning and memory deficits [5] as well as accelerated rate of cognitive decline in patients with mild dementia [6]. Furthermore, T2DM causes neuropathy and leads to poor quality of life by decreasing one’s ability to perform activities of daily living (ADLs) [1, 2] via increased the severity and progressive abnormalities in brain structures and cognitive function [7].

In addition to cognitive decline, recent clinical studies showed that the prevalence of depression was increased in the patients with T2DM [8, 9]. The studies showed that the symptoms of a major depressive episode included sadness combined with decreased energy, changes in thinking, and appetite changes [10], symptoms which may occur in T2DM patients with or without depression [11]. Accordingly, the studies reported that depression was associated with adverse effects for people suffering from T2DM, including impairment of glycemic control, eating habits, and exercise [12, 13]. However, the effects of depression on cognitive function and ability to perform ADLs in T2DM patients have never been investigated.

In addition to the impairment of cognition and ADLs, previous studies also found that the concentration of salivary alpha-amylase was increased under both physical stress, such as treadmill exercise, running, bicycle exercise [14] and psychological stress such as depression, and anxiety [15, 16]. Furthermore, it was established that the hyperglycemia and systemic inflammation in T2DM patients causes changes in the microvasculature and basal membrane of the salivary gland [17, 18], which lead to salivary dysfunction [17, 18]. Previous studies showed that salivary-alpha amylase activity decreased in diabetic rats, diabetic dogs [19, 20] and rats treated long-term with a high-fat diet [13]. Moreover, a recent clinical study demonstrated that low serum amylase levels were observed in patients with obesity, type 1 and 2 diabetes, and metabolic syndrome (MetS) [21]. However, the level of salivary alpha-amylase activity and its correlation with cognitive function or level of depression in T2DM patients with depression have never been investigated. All of those findings lead to our research hypotheses that 1) T2DM decreases the cognition and ability to perform ADLs as well as the salivary alpha-amylase activity; 2) T2DM patients with depression had higher level of these impairments than T2DM patient; and 3) the salivary alpha-amylase activity is correlated with cognition both in T2DM patients with and without depression.

## Materials and methods

The study protocol was reviewed and approved by the Institutional Ethics Committee of the Faculty of Associated Medical Sciences, Chiang Mai University, Chiang Mai, Thailand (Ethic number: AMSEC-62EX-010). All participants gave written informed consent for participation in this study. All methods were performed in accordance with the relevant guidelines and regulations. In this study, 130 participants aged 45–70 years, both male and female, were divided into two groups: 30 healthy people (control group) and 100 diabetic patients. Of these, 85 diabetic patients met eligibility criteria and were included in the study. The eligibility criteria used in this study included a diagnosis of type 2 diabetes by a medical doctor, at least 1 year of drug treatment for T2DM, no physical disability, and the ability to sit for 90 minutes for all study protocols. Level of depression was assessed for all participants using the Thai version of the Patient Health Questionnaire (PHQ-9) as a screening tool for major depression [22]. 85 diabetic patients were then divided into two groups: diabetic without depression (n = 50) and diabetic with depression (n = 35). Metabolic function, cognitive function, salivary-alpha amylase activity, and ADL function were determined for each patient in all three groups.

### Data collection

Researchers took history, sampled blood and saliva and assessed cognitive function, ADL, and depression level on the same day.

### Biochemical measurements

For the measurement of glycemic indices in diabetic patients, blood samples were collected after a minimum of 12 h of fasting since the last meal or snack in order to measure biomedical indicators including HbA1c and fasting blood glucose (FBG) [23]. Blood samples were collected by licensed medical technologists and measured at the clinical analysis laboratory.

### The cognitive function assessment

In this study, the Montreal Cognitive Assessment-Basic (MoCA-B) was used to detect cognitive impairment [24]. This questionnaire can detect mild cognitive impairment in individuals with limited education and can assess the different cognitive domains including visual perception, executive functioning, language, attention, memory, and orientation [24]. Test-retest reliability of MoCA-B was 0.91 (P < .001) and internal consistency was 0.82 [24]. The maximum score is 30 points; a score of 25 or above is considered normal. In this study, MoCA-B was administered by one occupational therapist and the time administered ranged between 15 and 20 min.

### The assessment of depression

The Thai version the Patient Health Questionnaire (PHQ-9) was used to detect depression in patients with T2DM [22]. The Thai version of the PHQ-9 has acceptable psychometric properties for screening for major depression [22]. In the present study, depression was assessed based on the PHQ-9 scores. The maximum score is 27 points; a total score of 0 to 4 points is classified as “normal,” 5 to 9 points as “mild depression,” and 10 to 27 points as “Moderate to severe depression” [22]. In this study, the group of diabetic patients with depression included both mild and severe depression levels. In this study, PHQ-9 was administered by one occupational therapist and the time taken to administer the assessment ranged from 10 to 15 min.

### Assessment of ability to perform activities of daily living (ADLs)

The Barthel Index (BI) is an assessment tool used to measure performance of the basic activities of daily living (B-ADL) or self-care. The BI assessment tool is divided into 10 items: feeding, personal toileting, bathing, dressing, grooming, controlling bladder, controlling bowel, transferring, mobility, and ascending and descending stairs [25]. The maximum score is 100 points; a total score of 0 to 20 points is classified as “total dependence”, 21 to 60 points as “severe dependence”, 61 to 90 points as “Moderate dependence”, and 91 to 99 points as “independence” [25]. In this study, BI was administered by one occupational therapist and the time taken to administer the assessment ranged from 10 to 15 min.

The Lawton Instrumental Activities of Daily Living Scale (I-ADL) is an assessment tool used to measure ability to perform more complex ADLs necessary for living in a community [26]. Because I-ADL function is usually lost before B-ADL function, I-ADL assessment can identify the decline of physical and cognitive functions in older adults [26]. The I-ADL assessment tool is divided into 8 items: using a telephone, shopping, preparing food, housekeeping, doing laundry, transportation, being responsible for one’s own medications, and handling finances [26]. The Lawton I-ADL scale scores each item as 0 (“less able”) or 1 (“more able”), so the summary score ranges from 0 (low functioning, dependent) to 8 (high functioning, independent) [26]. In this study, this assessment tool was administered by one occupational therapist and the time taken to administer the assessment ranged from 10 to 15 min.

### The assessment of salivary alpha-amylase activity

The saliva samples were collected in the morning. Participants were instructed not to eat, drink, or exercise before collection. To collect saliva samples, the passive drooling method was used [27]. Saliva was stored at −20°C until the analysis of salivary-alpha amylase activity [27]. The salivary-alpha amylase activity was measured by using a commercial salivary alpha amylase kinetic enzyme assay kit [28]. This kit was specifically designed and validated for the kinetic measurement of salivary-alpha amylase activity [28]. The salivary-alpha amylase activity then was detected by using microplate reader at 405 nm [28].

### Data analysis

All data were presented as mean value ± SEM. Comparisons were made using the one way ANOVA followed by the Fisher post-hoc test. Categorical variables were expressed as frequencies and comparison between groups was analyzed by using the Pearson χ^2^ test. The correlation between each factor with the salivary-alpha amylase activity was analyzed using the linear regressions. A *P* value < 0.05 was considered to be statistically significant.

## Results

### General characteristics of the control, T2DM, and T2DM with depression groups

The general characteristics of participants are shown in Table 1. Our data show that gender, age, education, and occupation had no significant differences among the three groups (Table 1). Additionally, the duration of T2DM and duration of T2DM treatment showed no significant difference between the T2DM with depression and T2DM without depression at the time of this study (Table 1). Also, the underlying diseases, such as hyperlipidemia and hypertension, had no significant differences among the three groups at the time of this study. Level of depression was determined by using a PHQ-9 screening. An increase of PHQ-9 score indicates an increase in the severity of depression [22]. Our results showed no significant difference in the level of depression between the T2DM without depression group and the control group (Table 1). The PHQ-9 screening also showed a significantly higher level of depression for the T2DM with depression group than for the control and T2DM without depression groups (p<0.05, Table 1).

**Table 1:** General characteristics of the control, type 2 diabetes, and type 2 diabetes with depression groups.

General characteristics	Control	T2DM without depression	T2DM with depression
**Gender (Frequency)**			
Male/Female	12/18	12/38	10/25
**Age (years)**			
Average (SD)	61.37±0.72	59.88±1.04	61.54±1.63
**Education (Frequency)**			
Uneducated/Educated	6/24	14/36	15/20
**Occupation (Frequency)**			
Unemployed/Employee	8/22	10/40	15/20
**Underlying disease (Frequency)**			
Yes/No	4/26	44/6*	32/3*
**T2DM Duration**			
Average (SD)	-	18.82±3.32	14.14±4.27
**Duration of T2DM treatment**			
Average (SD)	-	15.54±2.23	12.34±2.83
**PHQ9 score**			
Average (SD)	0.80±0.85	1.50±1.27	7.54±2.86*^,^^†^

T2DM: Type 2 diabetes, SD: The standard deviation, PHQ9: the Patient Health Questionnaire-9

* p < 0.05 vs. control group,

^†^ p < 0.05 vs. T2DM without depression

### The comparison of metabolic function between T2DM patients with depression and those without depression

Body weight, body mass index (BMI), fasting blood glucose (FBG) level, and HbA1c in both the T2DM with or without depression groups were significantly higher than the control group (p<0.05, Table 2). Interestingly, FBG level in the T2DM with depression group was significantly higher when compared with the T2DM without depression group (p<0.05, Table 2).

**Table 2:** Metabolic function of the control, type 2 diabetes, and type 2 diabetes with depression groups.

Glycemic indices	Control	T2DM without depression	T2DM with depression
**Weight (kg)**			
Average (SE)	68.53±1.18	77.42±1.41*	73.49±1.71*
**BMI (kg/m** ^**2**^ **)**			
Average (SE)	19.41±0.26	29.55±0.62*	27.48±0.71*
**Fasting blood glucose (mg/dl)**			
Average (SE)	90.77 ± 0.99	119.58±3.91*	155.94±11.04*^,^^†^
**HbA1c (%)**			
Average (SE)	5.23±0.03	7.54±0.03*	6.66±0.06*

T2DM: Type 2 diabetes, SE: The standard error; HbA1c: Hemoglobin A1C; BMI: Body mass index

* p < 0.05 vs. control group,

^†^ p < 0.05 vs. T2DM without depression

### The comparison of cognitive function between T2DM patients with depression and those without depression

**Fig 1:**
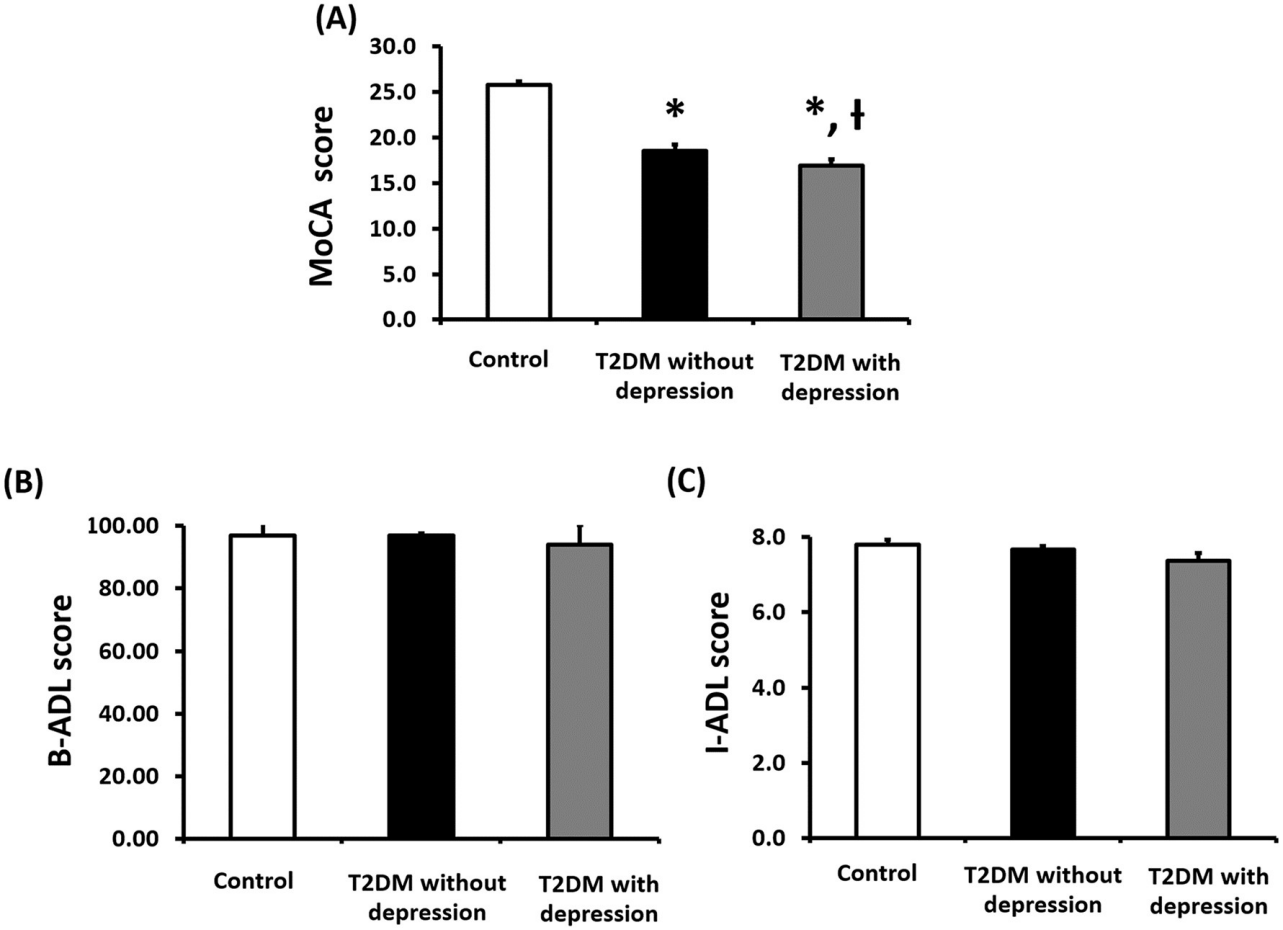
Effects of depression on the cognitive function and the ability to perform ADLs in T2DM patients. Effects of depression on the cognitive function (MoCA score (A)) and ability to perform ADLs (B-ADL score (B) and I-ADL score (C) in T2DM patients. * p < 0.05 vs. control group, † p < 0.05 vs. T2DM without depression group. Abbreviation: MoCA = Montreal cognitive assessment, B-ADL = Basic activities of daily living, I-ADL = Lawton instrumental activities of daily living, T2DM = Type 2 diabetes.

In this study, cognitive function was determined by using the MoCA screening and was represented in the data as a MoCA score, with 25 or above considered normal cognitive function [24]. Thus, the decreased MoCA score represents the decline of cognitive function. Our results showed that the MoCA scores of both T2DM group with or without depression group were significantly lower than the control group (p<0.05, Fig 1A). Interestingly, the MoCA scores in the T2DM patients with depression was significantly lower than T2DM patients without depression (p<0.05, Fig 1A).

In this study, ADL function was divided into two categories including basic ADLs (B-ADL) and instrumental ADLs (I-ADL). Our results show that the B-ADL function scores in both T2DM with or without depression groups were not significantly different when compared with control group (Fig 1B). In addition, our results demonstrated that I-ADL function scores in both T2DM with or without depression groups were not significantly different when compared with control group (Fig 1C). All of these findings indicated that depression did not affect the impairment of ability to perform ADLs in T2DM patient.

### The comparison of salivary alpha-amylase activity between T2DM patients with depression and those without depression

**Fig 2:**
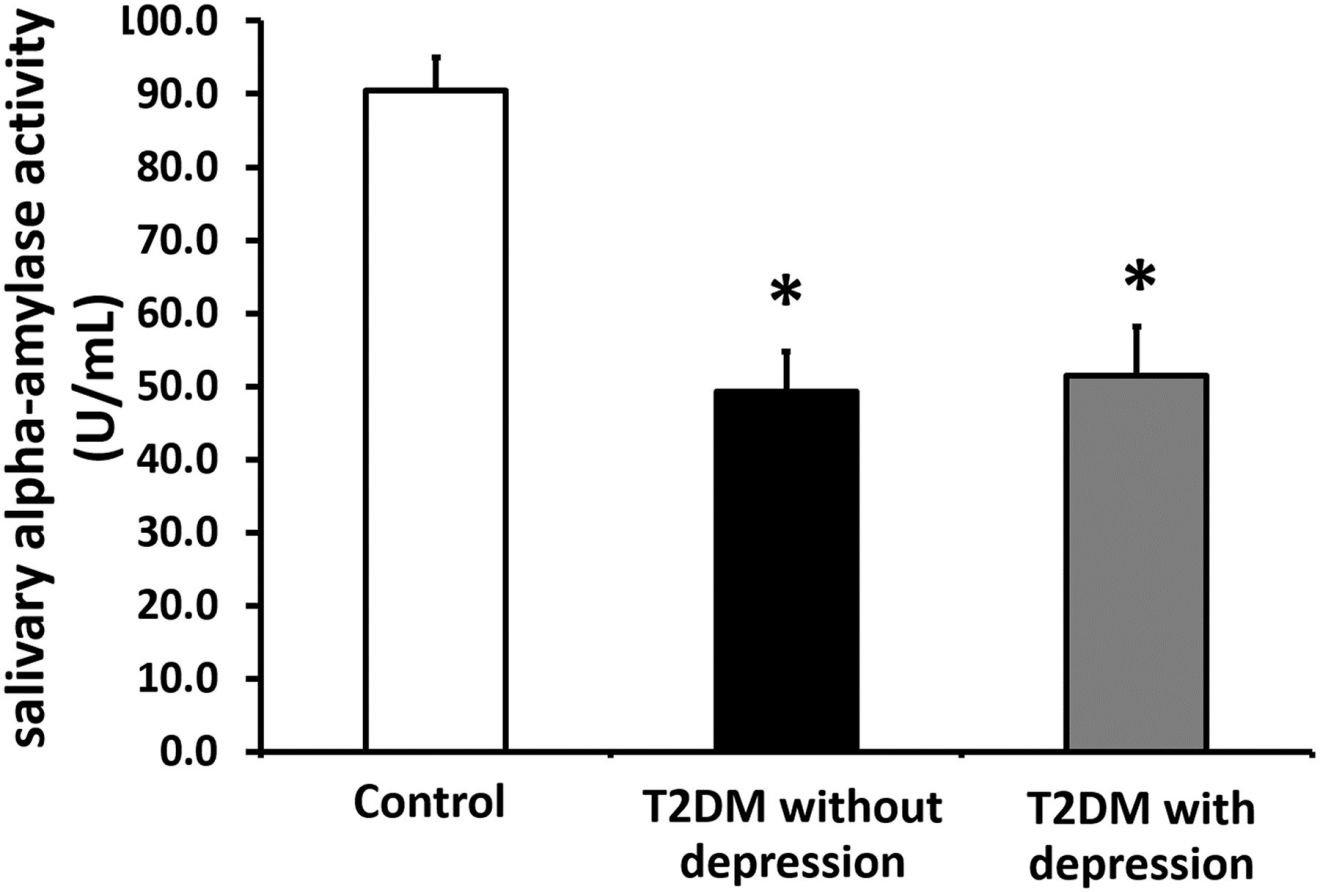
Effect of depression on salivary alpha-amylase activity in T2DM patients. * *p* < 0.05 vs. control group, T2DM = Type 2 diabetes.

Our results show that salivary alpha-amylase activity in both T2DM with or without depression groups were significantly decreased when compared with control group (p<0.05, Fig 2). However, the levels of salivary alpha-amylase activity in the T2DM patients with depression were not significantly different when compared with T2DM patients without depression (Fig 2). All of these findings indicated that depression did not affect the decrement of salivary alpha-amylase activity in T2DM patient.

### The correlation between salivary alpha-amylase activity, MoCA score and PHQ-9 score

In this study, the associations between salivary alpha-amylase activity and cognitive function or depression level were assessed by linear regression analysis. The FBG, BMI, age, and gender, which can influence the change of salivary-alpha amylase activity, were considered as covariates. Our result showed that MoCA scores and PHQ-9 scores were not correlated with salivary-alpha amylase activity in control group (Table 3). However, the result in the T2DM patients without depression showed that MoCA score, but not PHQ-9 score, was positively associated with salivary-alpha amylase activity. On the other hand, PHQ-9 score was positively correlated with salivary-alpha amylase activity in T2DM patients with depression while there was no association between MoCA score and salivary-alpha amylase activity in this group. All of these findings suggested that cognitive function was associated with the salivary-alpha amylase activity in T2DM patients without depression, while the depression was associated with the salivary-alpha amylase activity in T2DM with depression.

**Table 3:** Correlation between salivary-alpha amylase activity and MoCA and PHQ-9.

Variables	Control group			T2DM without depression			T2DM with depression		
	B	95%CI	p-value	B	95%CI	p-value	B	95%CI	p-value
**MoCA score**									
Amylase	-1.310	(-6.711, 4.092)	NS	**3.592**	**(2.219, 4.964)**	**<0.01**	-0.147	(-3.885, 3.591)	NS
Amylase + covariates	-1.570	(-7.303, 4.163)	NS	**3.329**	**(1.850, 4.807)**	**<0.01**	-0.207	(-4.349, 3.936)	NS
**PHQ-9 score**									
Amylase	6.715	(-4.446, 17.876)	NS	6.630	(-2.114, 15.374)	NS	**9.732**	**(6.242, 13.222)**	**<0.01**
Amylase + covariates	6.284	(-6.591, 19.159)	NS	4.461	(-4.197, 13.119)	NS	**9.793**	**(6.151, 13.436)**	**<0.01**

T2DM: Type 2 diabetes; MoCA: Montreal Cognitive Assessment; PHQ9: the Patient Health Questionnaire-9

Covariates = body mass index, age, and fasting blood glucose

## Discussion

The major findings of this study are as follows: 1) the metabolic disturbance and cognitive decline were observed in T2DM patients; 2) T2DM patients with depression had higher hyperglycemia and cognitive decline levels than those without depression; 3) both T2DM patients with and without depression did not impair activities of daily living (ADLs); 4) both T2DM patients with and without depression equally decreased salivary-alpha amylase activity; and 5) cognitive function was correlated with salivary-alpha amylase activity in T2DM patients without depression, while depression was correlated with salivary-alpha amylase activity in T2DM patients with depression.

In this study, the general characteristics within each group showed no significant difference from the other groups. Metabolic dysfunctions of the T2DM patients with and without depression were significantly higher than those of healthy people, as indicated by increased body weight, BMI, fasting blood glucose (FBG), and HbA1c. Consistency, several previous studies also demonstrated that metabolic dysfunction was also observed in T2DM patients [29, 30]. Interestingly, FBG levels in the T2DM patients with depression were significantly higher than those of T2DM patients without depression. This is consistent with the previous studies showing that depression was associated with hyperglycemia and glycemic disturbance in patients with T2DM [31, 32]. It is possible that the increase of mental stress can increase blood glucose levels in depressed patients due to poor self-care behaviors. This is consistent with previous studies that showed that depressive symptoms and mental stress were associated with poor diet, low levels of physical activity, and high levels of blood glucose [31, 32]. The previous study also showed that depression correlated with metabolic diseases and diabetes involving pro-inflammatory cytokine and insulin resistance [33]. Therefore, the higher level of hyperglycemia in T2DM patients with depression may be caused by the induction of mental stress and poor self-care behaviors during depression.

Regarding cognitive function, our results demonstrated that cognitive function in the T2DM patients with and without depression was significantly lower than that of healthy people. Decline of cognitive function has been observed in patients with diabetes [34]. Similarly, another study suggested that T2DM is associated with an impairment of episodic memory and decreased executive function [6]. Interestingly, the decline of cognitive function in the T2DM patients with depression was significantly increased when compared with T2DM patients without depression. This finding is supported by the previous studies showing that depression is a risk factor for MCI and promotes the development of MCI into dementia [35, 36]. Moreover, the depression was shown to increase cognitive decline via reducing synaptic plasticity and increasing pro-inflammatory cytokines [37]. Thus, it is possible that depression may affect cognitive impairment in T2DM patients.

ADLs are routine activities people do every day, and ability to perform ADLs is one of the factors that contribute to quality of life [38]. It has been established that one of the worst effects of diabetes was the cognitive decline that can lead to ADL disability [1, 2]. Thus, reduction of cognitive decline should increase the ability to perform ADLs. Although our results demonstrated that T2DM with or without depression increased the levels of cognitive impairment, the ability to perform ADLs in these patients showed no change when compared to that of healthy people. It is possible that the severity of cognitive impairment in our patients with T2DM may not be high enough to reach to a critical threshold level for the impairment of ADL activity. Thus, no change of ADL function scores in T2DM patients with or without depression was found in this study. This possibility is consistent with previous studies suggesting that the optimal level of cognition function for completing ADLs can be observed in patients with dementia and MCI [39, 40]. So, it is possible that the severity of cognitive impairment of T2DM patients in this study was not enough to trigger an impairment of ADL function, when compared to healthy people.

In this study, we studied the activity of salivary α-amylase in T2DM patients with and without depression. Our results demonstrated that salivary-alpha amylase activity in T2DM patients without depression was significantly lower than that of healthy individuals. This finding corresponds with animal studies showing that salivary α-amylase activity decreased in diabetic rats and diabetic dogs [19, 20]. Moreover, we investigated the salivary α-amylase activity of T2DM patients with depression. Previous studies have shown that salivary α-amylase was increased under both physical stress, such as treadmill exercise, running, bicycle exercise, cold exposure [41] and psychological stress such as depression, and anxiety [15, 16].

Our study is the first study demonstrated that salivary-alpha amylase activity in T2DM patients with depression was significantly lower than that of healthy people. However, our results showed that salivary-alpha amylase activity in the T2DM patients with depression showed no difference when compared with T2DM patients without depression. It is possible that a decline of salivary-alpha amylase activity in our study was caused by diabetic condition. In the future study, the mechanistic insight of the decline of salivary-alpha amylase activity in the T2DM patients needs to be further investigated.

We also found that cognitive function and the level of depression associated with salivary-alpha amylase activity in all T2DM patients. However, we found that only cognitive function was positively associated with the salivary-alpha amylase activity in T2DM patients without depression, while the depression level was not associated with the salivary-alpha amylase activity in T2DM patients without depression. These results were adjusted with FBG, BMI, and age. Thus, we suggested that salivary-alpha amylase activity was independently associated with cognitive function in T2DM patients without depression. Regarding T2DM patients with depression, the results demonstrated that only depression level was associated with salivary-alpha amylase activity. This finding is consistent with the studies showing that the elevated salivary-alpha amylase level was associated with depression severity in the major depressive disorder patients [42, 43]. According to other studies, elevated secretion of salivary-alpha amylase was associated with a higher prevalence of psychiatric disorders [15, 27]. Thus, we suggested that salivary-alpha amylase activity was correlated with depression in T2DM patients with depression in this study.

## Conclusion

T2DM patients with depression had higher hyperglycemia and cognitive decline levels than those without depression. Both T2DM patients with and without depression equally decreased salivary-alpha amylase activity. Moreover, we found that cognitive function was associated with the salivary-alpha amylase activity in T2DM without depression, while the severity of depression was associated with the salivary-alpha amylase activity in T2DM patients with depression.

## Study limitation

The results of our study are valid only for the Thai population and may not extent to other countries.

## Supporting information

S1 File The supporting information file in this study. (XLSX)
